# Low frequency of defective mismatch repair in a population-based series of upper urothelial carcinoma

**DOI:** 10.1186/1471-2407-5-23

**Published:** 2005-03-01

**Authors:** Kajsa M Ericson, Anna P Isinger, Björn L Isfoss, Mef C Nilbert

**Affiliations:** 1 Departments of Oncology, University Hospital, Lund, Sweden; 2 Departments of Pathology, University Hospital, Lund, Sweden

## Abstract

**Background:**

Upper urothelial cancer (UUC), i.e. transitional cell carcinomas of the renal pelvis and the ureter, occur at an increased frequency in patients with hereditary nonpolyposis colorectal cancer (HNPCC). Defective mismatch repair (MMR) specifically characterizes HNPCC-associated tumors, but also occurs in subsets of some sporadic tumors, e.g. in gastrointestinal cancer and endometrial cancer.

**Methods:**

We assessed the contribution of defective MMR to the development of UUC in a population-based series from the southern Swedish Cancer Registry, through microsatellite instability (MSI) analysis and immunohistochemical evaluation of expression of the MMR proteins MLH1, PMS2, MSH2, and MSH6.

**Results:**

A MSI-high phenotype was identified in 9/216 (4%) successfully analyzed patients and a MSI-low phenotype in 5/216 (2%). Loss of MMR protein immunostaining was found in 11/216 (5%) tumors, and affected most commonly MSH2 and MSH6.

**Conclusion:**

This population-based series indicates that somatic MMR inactivation is a minor pathway in the development of UUC, but tumors that display defective MMR are, based on the immunohistochemical expression pattern, likely to be associated with HNPCC.

## Background

Upper urothelial carcinomas (UUC) represent about 5% of the urinary tract tumors, with transitional cell carcinomas of the renal pelvis and the ureter being the most common [1]. Exogenous agents such as smoking and occupational exposures to e.g. acrylamines constitute risk factors that are estimated to cause up to half of the tumors [2]. Hereditary factors also contribute to the development of UUC with a 2-fold increased risk among first-degree relatives [3]. The familial cases develop due to site-specific inheritance as well as within the hereditary nonpolyposis colorectal cancer (HNPCC) syndrome [1,3-5]. Individuals with HNPCC are at increased risk for several types of cancer, with the highest life-time risks for colorectal cancer (80%), endometrial cancer (40–60%), ovarian cancer (10–15%), cancer of the small intestine and upper urothelial cancer [4], and the revised Amsterdam criteria for the diagnosis of HNPCC consider these tumor types to be associated with the syndrome [6]. Although HNPCC-patients have a 14 to 75-fold increased risk of UUC, with the highest risks reported for carriers of mutations in *MSH2*, the absolute lifetime risk for mutation carriers to develop UUC is <10% [7-9].

HNPCC is caused by a germline mutation in a DNA mismatch-repair (MMR) gene, most commonly affecting either of the genes *MLH1 *(40%), *MSH2 *(50%) or *MSH6 *(10%) [10,11]. Over 95% of the HNPCC-tumors are characterized by wide-spread microsatellite instability (MSI) and 90% by loss of immunohistochemical expression of the MMR protein affected [12]. Hence, these analyses are used in the clinical diagnosis of suspected HNPCC cases. However, somatic MMR defects occur in a subset of certain sporadic tumor types, e.g. in 15–20% of gastrointestinal and endometrial cancer, and are in most of these tumors caused by somatic hypermethylation of the *MLH1 *promoter [13,14].

Studies of the contribution of defective MMR to the development of urothelial carcinomas, assessed using MSI analysis, loss of MMR protein expression, and MMR gene mutations, have found a low frequency (<10%) of MMR defects in urothelial carcinomas of the urinary bladder [15], but have indicated a high frequency (15–45%) of MMR defects in UUC [16-19]. Since data on the frequency of MMR defects in UUC are scarce and in order to characterize the contribution of the different MMR proteins to development of UUC, we assessed MSI and immunohistochemical expression of MLH1, MSH2, and MSH6 in a population-based series of UUC.

## Methods

### Patient Material

In Sweden a population-based national Cancer Registry was started in 1958 and applies mandatory registration by both clinicians and pathologists in order to achieve maximal coverage (estimated to be 98%). We applied the southern Swedish part of the registry, which currently contains about 300.000 entries, to identify all carcinomas of the upper urothelial tract that had developed between 1992 and 1999. We identified 262 patients with a median age of 70 (range 34–90) years and a male:female ratio of 1.8:1. For further analyses, 27 patients were excluded because of lack of tumor blocks, and 19 because of autopsy-based diagnosis with autolysis that prevented good quality immunostaining. Hence, 216 patients with a median age of 69 (range 34–89) years were analyzed. Tumor location was as follows for the whole material (cases analyzed within parenthesis): renal pelvis 173 (154), ureter 75 (60) and an unspecified tumor location in 14 (2) patients. Data on family history of cancer or blood samples for mutation analysis were not available since the study was retrospective and register-based. Ethical approval for the study was obtained from the ethics committee at Lund University.

### Analysis of microsatellite instability (MSI)

Representative tumor blocks containing at least 20% tumor tissue were selected and DNA was extracted from 3 × 10-μm sections of formalin-fixed, paraffin-embedded tissue through incubation of the samples in EDTA-Tris-buffer with Proteinase K at 65°C for at least 2 hours, followed by boiling, centrifugation, and removal of the aqueous phase, which was stored at 4°C. MSI was assessed with the National Cancer Institute (NCI) panel; BAT25, BAT26, BAT34, BAT40, D2S123 and D5S346 [20]. These markers identify MSI with high accuracy in colorectal cancer, but the sensitivity of individual markers may vary between different tumor types [21]. The primer sequences used have been reported previously [22]. The markers were fluorescencely-labeled as follows: NED™ (yellow) for BAT25, 6-FAM™ (blue) for BAT26, BAT34C4 and D2S123, and HEX™ (green) for BAT40 and D5S346. The DNA microsatellite sequences were amplified by PCR according to the following programme; 94°C for 7 minutes, 10 × (94°C for 15 seconds, 45°C (BAT 25) / 50°C (other markers) for 15 seconds, and 72°C for 15 seconds), 23 × (89°C for 15 seconds, 45°C / 50°C for 15 seconds, and 72°C for 15 seconds), 72°C for 7 minutes, followed by a final cooling step at 4°C. 0.5–2 μl PCR product was mixed with 12 μl deionized formamide (Hi-Di Formamide, Applied Biosystems) and 0.5 μl ROX™ 500 Size Standard (Applied Biosystems), denatured at 95°C for 2 minutes, chilled on ice, and separated in Performance Optimized Polymer-4 (POP-4™) on the ABI PRISM™ 3100 Genetic Analyzer (Applied Biosystems) for fragment analysis. MSI was defined by the presence of extra peaks demonstrating altered length of the repetitive sequence. Data from at least three markers were required for the classification of tumors as microsatellite stable (MSS). The tumors were regarded as MSI-high if at least two microsatellites showed instability and as MSI-low if only one marker showed instability. All cases with suspected MSI were verified through repeated analysis.

### Immunohistochemistry

Immunohistochemical staining was performed using 4-μm sections of formaline fixed, paraffin-embedded tissue, which were mounted on DAKO ChemMate Capillary Gap Microscope Slides (DAKO A/S BioTek Solutions, USA) and dried at room temperature overnight followed by incubation at 60°C for 1–2 hours. The tissue sections were deparaffinized in xylol and rehydrated through descending concentrations of alcohol. Antigen retrieval was achieved by microwave-treatment in 1 mM EDTA, pH 9.0, at 900 W for 8 minutes followed by 15 minutes at 350 W. The slides were then allowed to cool for at least 20 minutes in the EDTA-solution. Immunohistochemical staining was performed in an automated immunostainer (TechMate 500 Plus, DAKO), according to the manufacturers instructions. The main steps were as follows: Mouse monoclonal IgG antibodies to MLH1 (clone G168-15, dilution 1:100, PharMingen, San Diego, CA, USA) MSH2 (clone FE-11, dilution 1:100, Oncogene research products, Boston, MA, USA), MSH6 (clone 44, dilution 1:1000, BD Transduction Laboratories) and PMS2 (clone:A16-4, dilution 1:500, BD Pharmingen) were applied and the sections were incubated at room temperature for 25 minutes.

**Figure 1:**
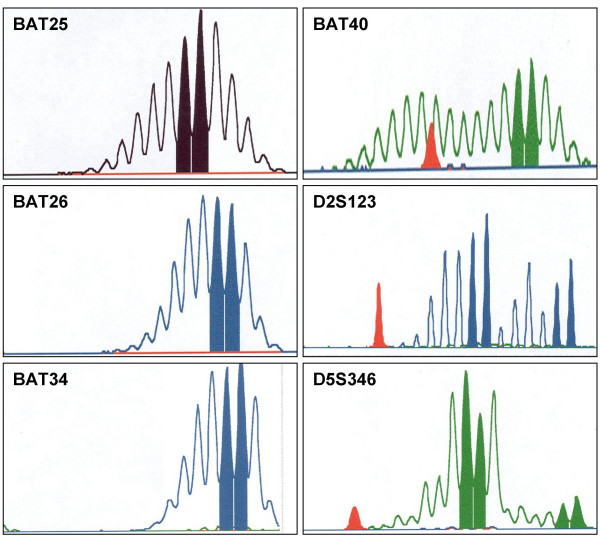
Microsatellite instability in case RP8-626 where the MSI-curves show instability with 1–2 additional peaks for the markers BAT25, BAT26 and BAT34 and larger size variation for the markers BAT40, D2S123 and D5S346.

Thereafter, the slides were incubated with biotinylated anti-mouse antibody (DAKO) for 25 minutes (for MLH1 and MSH2) or with rabbit anti-mouse immunoglobulins (DAKO, dilution 1:400) for 20 min (for MSH6 and PMS2). Endogenous peroxidase activity was blocked in Peroxidase-blocking solution (DAKO) for 3 × 2,5 minutes. This was followed by incubation with streptavidin-horseradish peroxidase for 25 minutes for MLH1 and MSH2, whereas EnVision™/HRP rabbit/mouse (DAKO) incubation for 25 min was used for MSH6 and PMS2. Finally, the tissue sections were treated with diaminobenzidine (DAB) for 3 times 5 min, counterstained with hematoxylin for 1 min, rinsed in running tap water for 10 min, dehydrated in ascending concentrations of alcohol and mounted. After each step, the sections were rinsed in Tris buffered saline, pH 7.4, and Tween-20. In order to block nonspecific protein binding, bovine serum albumin was added to the buffer before the antibody incubation steps in the MLH1 and MSH2 stainings. A detailed protocol is available from the authors upon request. Two of the authors (K.E. and M.N.), who were blinded regarding the MSI status, independently evaluated all stained sections. Sections without nuclear staining in the tumor cells, in the presence of normal nuclear staining in lymphocytes and normal epithelial or stromal cells in the same section, were considered to have a lost expression (Fig. 1). The expression was classified as present, absent or non-evaluable without grading of the staining intensity.

## Results

### Microsatellite analysis

For the MSI analysis of the 216 cases, 16 tumors were excluded because of small tumor size or less than 20% tumor tissue in the samples, and 6 tumors were excluded because of lack of information from at least 3 MSI markers, which left 194 tumors successfully analyzed. A MSS phenotype was identified in 180 tumors, MSI-low in 5, and MSI-high in 9 tumors (table 1, figure 1).

### Immunohistochemistry

**Figure 2:**
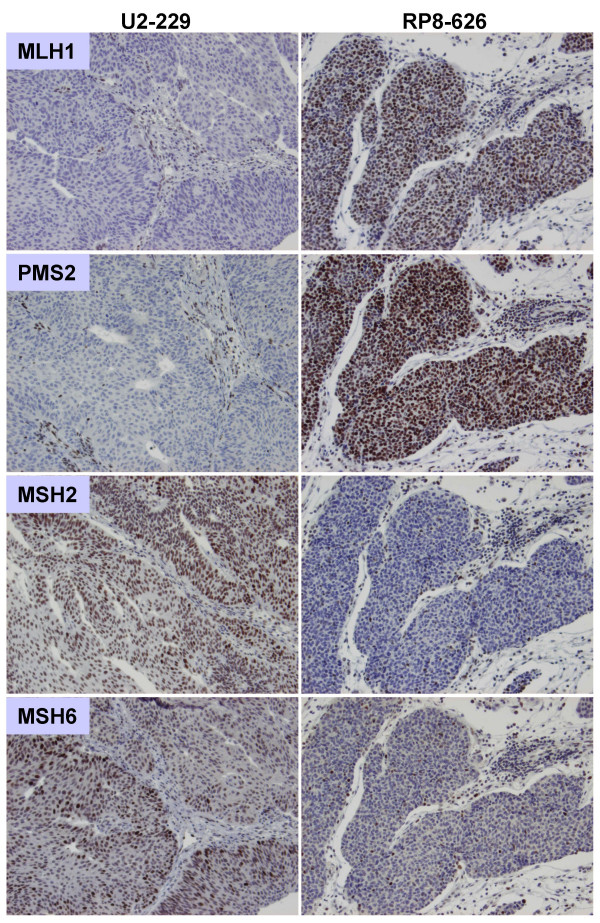
Immunohistochemical staining for the MMR proteins shows loss of nuclear expression for MLH1 and PMS2 and retained expression of MSH2 and MSH6 in case U2-229, and loss of MSH2 and MSH6 with retained expression of MLH1 and PMS2 in case RP8-626.

Immunohistochemical staining for the MMR proteins gave evaluable results for MLH1 in 211 tumors, MSH2 in 216, MSH6 in 200 tumors and PMS2 in 215 tumors. Of the 180 MSS tumors, 177 showed retained expression for all evaluable proteins, as did also the 5 MSI-low tumors. One MSS tumor that was not assessed for MSI due to a small amount of tumor material, showed loss of MSH2 and MSH6 expression, one MSS tumor showed loss of MLH1 and PMS2, one MSS tumor showed loss of MSH6 expression, and one MSS tumor showed loss of MSH2 expression. Among the 9 MSI-high tumors, 5 showed a concomitant loss of expression of MSH2 and MSH6, 1 tumor showed loss of expression of MSH6 and 1 tumor showed loss of expression of MLH1 and PMS2. Retained expression of all four proteins was found in 2 MSI-high tumors of the renal pelvis (table 1, figure 2).

### Synchronous and metachronous tumors

Eleven patients had developed synchronous tumors of the upper urinary tract, and these cases were all analyzed. In one patient with synchronous urethral tumors the tumor tissue showed MSI and loss of expression of MLH1 in both tumors (U2-229), and the other patients with synchronous tumors had MSS tumors all of which showed retained expression of all three MMR proteins (table 1). Metachronous UUC occurred in 3/262 patients, 2 of whom were included in the series analyzed, and these tumors were MSS and MSI-low, respectively, but both tumors showed retained MMR protein expression.

In the whole series, 122 (97 among the analyzed cases) patients had been diagnosed with another malignancy, which was bladder cancer in 67 cases (54 among the cases analyzed). Among the cases with MSI and/or immunohistochemical MMR protein loss, 8 metachronous tumors developed and 5/5 analyzed (a leiomyosarcoma, a colon tumor, an endometrial cancer and 2 bladder tumors) displayed MSI and immunohistochemical loss of the concordant MMR protein (table 1).

## Discussion

Urothelial carcinomas of the upper and the lower urothelial tract share many clinical and epidemiological traits. However, the UUC have specifically been associated with HNPCC, and in line with this observation the contribution of defective MMR has been reported to differ between these tumor types. In bladder cancer, a MSI-high phenotype has been found in 3–10% of the tumors [15,23], whereas elevated microsatellite alterations at selected tetranucleotides (EMAST) has been described at a higher rate in bladder cancer and the latter phenomenon is being perused as a tumor marker in urine [24]. Higher MSI rates have been reported in UUC with 13–31% of the tumors showing MSI [16-19]. A similar anatomical specificity has been described in the ventricle with a higher number of MSI tumors in the antrum, and in the colorectum with 20% MSI tumors in the cecum and <5% in the rectum [25-27].

We applied the population-based southern Swedish Cancer Registry to assess the contribution of defective MMR to the development of UUC. The results are based on 216/262 (82%) of the tumors that occurred in the southern Sweden health care region between 1992 and 1999. A MSI-high phenotype was found in 9/216 (4%) patients and a MSI-low phenotype in 5/216 (2%). In 11/216 cases synchronous tumors occurred within the urothelial tract and 1 patient (U2-229) had synchronous MSI-high tumors, all of which displayed a concordant immunohistochemical loss of MLH1. Thus, the vast majority of synchronous UUC does not display MMR defects and does not develop within the HNPCC syndrome. The overall frequency of MSI tumors detected in our study, 4%, is lower than the 13–31% previously reported (table 2) [16-19]. Possible reasons for the discrepancy include that our study was unselected and population-based. Furthermore, Müller *et al*. [28] have suggested that microsatellite instability analysis should optimally be performed by using microdissection, where analysis is made on DNA extracted from tumor cells without dilution of DNA from normal cells. However, this was not available at our institution at the time the study. The marker selection is probably not the cause of discrepancy since the NCI marker panel for MSI analysis has proven effective in several extracolonic tumor types such as endometrial, ovarian and gastric cancer [20,29]. Hartmann et al. [18] identified BAT40 (93% detection rate) and BAT25 (53%) as the best markers for the detection of MSI also in UUC and indeed reported that using a combination of the markers BAT40, BAT25, and BAT26 allowed identification of all MSI tumors. Whereas our finding of 5% MMR defects in renal pelvis tumors is in accordance with the 5–8% previously reported, we identified MMR defects in a lower (4%) fraction of the urethral tumors than the 25–41% previously reported [16-19,23].

Loss of immunostaining was in our series detected in 7/9 MSI-high tumors, in one tumor biopsy that was to small to allow MSI analysis, and in 2 MSS tumors (figure 2). The immunohistochemical expression loss affected MSH2/MSH6 in 6 cases, MSH2 in one, MSH6 in two, and MLH1/PMS2 in two cases. Concordant loss of the same MMR protein was observed in the patient who had developed multiple synchronous tumors (table 1). This frequency of immunohistochemical loss of expression in MSI-high tumors is similar to that previously reported (table 2) and thus demonstrates that loss of immunostaining for at least one of the MMR proteins investigated is found in about 85% of MSI-high UUC [18,23]. Regarding histological grade and stage among the tumors with MSI and/or MMR protein expression loss, the majority of the tumors were moderate differentiated (WHO-grade 2–3) and of early stages (table 1).

Synchronous/metachronous tumor development is common in urothelial cancer, mainly through intraepithelial migration or intraluminal dispersion of tumor cells [30]. In our series, 54/216 (25%) patients had developed metachronous bladder tumors. An increased incidence of metachronous tumors has been observed in patients with MMR defective UUC [19], and synchronous UUC is found in 1–2% of UUC patients [31]. Of the 8 patients with MMR defective tumors in our study, 5 had developed other malignant tumors, including two cancers of the urinary bladder, one colon cancer, one rectal cancer, one endometrial cancer, one cervical cancer, one soft tissue sarcoma, and one patient who had developed myelofibrosis (table 1). Among these tumors, 5 could be retrieved and were immunohistochemically stained. The leiomyosarcoma, the colon tumor, the endometrial cancer and 2 bladder tumors showed loss of expression for MSH2/MSH6, which suggests an association with HNPCC. About 1/3 of HNPCC patients develop metachronous primary tumors, and the concordant MSI and loss of MMR protein expression in these cases strongly suggests HNPCC, although mutation analysis was not performed.

The lifetime risk of developing UUC in HNPCC mutation carriers is estimated to be 4–10%, and UUC is in the revised Amsterdam criteria considered to be a HNPCC-associated tumor type, and screening for UUC is generally recommended in HNPCC-families (), with sonogrophy and urinary analysis. None of the patients in this series with MSI and/or IHC loss of MMR protein expression are previously known HNPCC patients in our health care region. Mutation analysis is not planned. A higher frequency of extraintestinal tumors has been reported in families with germline mutations in *MSH2*, and from the data available, *MSH2 *seems to play a predominant role also in UUC; loss of MSH2 expression has been reported in 33–60% of MSI-high UUC tumors [18,19,32,33]. Although our data suggest that MMR defects represent a minor tumorigenic pathway in the development of UUC, the high frequency of MSH2/MSH6 loss in MMR-defective tumors should caution clinicians to obtain an individual and a family history of cancer in patients with carcinomas of the renal pelvis and the ureter.

## Competing interests

The author(s) declare that they have no competing interests.

## Authors' contributions

KE conceived of the study, carried out the MSI analysis, performed immunohistochemical validation and drafted the manuscript. AI also carried out the MSI analysis. BI performed immunohistochemical validation. MN conceived of the study, and participated in its design and coordination and helped to draft the manuscript.

## Pre-publication history

The pre-publication history for this paper can be accessed here:



## Figures and Tables

**Table 1:** Clinicopathological data of MMR-defective tumors

Case no.	Sex / Age	Tumor location (Synchron./Metachron.)	WHO-Grade	Stage	Instable markers	Immunohistochemical expression			
						MLH1	MSH2	MSH6	PMS2
									

U1-113	F/54	ureter	G2	pTX	NE	+	-	-	+
	53	endometrium (M)			4/6	+	-	-	+

U2-229	F/57	ureter	G2	pT1	4–5/6	-	+	+	-
	57	ureter (S)			4–5/6	-	+	+	-

U3-821	M/61	ureter*	G2	pT1	2–3/6	+	-	-	+

RP1-101	M/60	renal pelvis	G2	pT3	3/5	+	-	-	+
	43	sarcoma (M)			3/4	+	-	-	+
	53	colon (M)			1/6¤	+	-	-	+

RP2-131	M/75	renal pelvis	G3	pT4	2/6	+	+	+	+

RP3-119	F/69	renal pelvis	G3	pTa	0/6	-	+	NE	-

RP4-267	F/67	renal pelvis	G2	pT1	5/5	+	-	-	+
	70	urinary bladder (M)			3/4	+	-	-	+

RP5-401	M/56	renal pelvis	G2	pTa	0/6	+	+	-	+

RP6-528	F/87	renal pelvis	G2	pT1	2/5	+	+	+	+
	87	urinary bladder (S)			0/6	+	+	+	+

RP7-613	F/57	renal pelvis^	G2	pT1	3/6	+	+	-	+
	54	urinary bladder (M)			4/6	+	-	-	+

RP8-626	M/66	renal pelvis	G3	pT2	6/6	+	-	-	+

RP9-701	F/49	renal pelvis#	G2	pTa	0/5	+	-	NE	+

RP10-809	F/82	renal pelvis	G3	pT4	4/4	+	-	-	+
									

¤Positive for BAT26

* Myelofibrosis at the age of 67 years.

^Cervical cancer at the age of 33.

# Rectal tumor at the age of 38.

G2 moderately differentiated

G3 poorly differentiated

**Table 2:** Summary of MMR studies in UUC

Reference	Sex (male: female)	Median age total/MSI-H	Total no. of patients (renal pelvis/ureter/multifocal)	Fraction MSI-high	IHC loss
Hartmann et al.	2.2:1	66 / 62	73 (40/22/11)	0.21	0.87
Catto et al.	2.0:1	68 / 66	71 (44/25/2)	0.13	0.78
Blaszyk et al.	2.8:1	70 / -	114 (61/53/-)	0.31	ND
Roupert et al.	3.4:1#	- / 70	164 (ND)	0.16	0.6*
Amira et al.	1.6:1	68 / 72	24 (19/4/1)	0.25	ND
Ericson et al.	1.8:1	67 / 67	216 (154/60/2)	0.04	0.78

* Only MSH2 assessed, ND: not determined

#Applies to subset with MSI
